# Cross-cultural adaptation and validation of the 3D-CAM Chinese version in surgical ICU patients

**DOI:** 10.1186/s12888-020-02544-w

**Published:** 2020-03-24

**Authors:** Dong-Liang Mu, Pan-Pan Ding, Shu-Zhe Zhou, Mei-Jing Liu, Xin-Yu Sun, Xue-Ying Li, Dong-Xin Wang

**Affiliations:** 1 Department of Anesthesiology and Critical Care Medicine, Peking University First Hospital, Beijing, China; 2 Department of Geriatric Psychiatry, Peking University Sixth Hospital, Beijing, 100191 China; 3 Department of Biostatistics, Peking University First Hospital, Beijing, China

**Keywords:** Delirium, Diagnosis, Screening tool, 3D-CAM, Chinese version, validation, Chinese

## Abstract

**Background:**

Accurate diagnosis of delirium is very important for prevention and treatment. Present study was designed to validate the 3-Minute Diagnostic Interview for CAM-defined Delirium Chinese version (3D-CAM-CN) in surgical ICU patients.

**Methods:**

In this prospective diagnostic study, the 3D-CAM was translated into Chinese with culture adaption. Two interviewers (Roles A and B) independently administrated 3D-CAM-CN assessment in adult patients from postoperative days 1 to day 3. At the meantime, a panel of psychiatrists diagnosed delirium according to the Diagnostic and Statistical Manual of Mental Disorders-fifth edition as the reference standard. The sensitivity and specificity were calculated to analyze the diagnostic character of the 3D-CAM-CN. *Kappa* coefficient was used to evaluate interrater reliability.

**Results:**

Two hundred forty-five adult patients were assessed for at least 2 days, resulting a total of 647 paired-assessments. When compared with the reference standard, the sensitivity and specificity of the 3D-CAM-CN assessment were 87.2 and 96.7%, respectively, by Role A and 84.6 and 97.4%, respectively, by Role B, with good interrater reliability (*Kappa* coefficient = 0.82, *P* < 0.001). It also performed well in patients with mild cognitive impairment, with the sensitivity from 85.7 to 100% and the specificity from 95.7 to 96.4%.

**Conclusion:**

Our results showed that the 3D-CAM-CN can be used as a reliable and accurate instrument for delirium assessment in surgical patients.

**Trial registration:**

This trail was approved by the Clinical Research Ethic Committee of Peking University First Hospital (No. 2017–1321) and registered on Chinese clinical trial registry on July 6, 2017 (ChiCTR-OOC-17011887).

## Background

According to the Diagnostic and Statistical Manual of Mental Disorders-fifth edition (DSM-5), delirium is a transient brain syndrome that develops over a short period of time and is characterized by fluctuating disturbances in attention, awareness and cognition [1]. The reported incidence varies from 11.2 to 23.0% in general patients and is up to 50.6% in those admitted to the intensive care unit (ICU) after major surgery [2–4]. The occurrence of delirium is associated with worse outcomes, including prolonged lengths of stay in ICU and hospital, increased postoperative complications and in-hospital mortality, as well as shortened long-term survival and worsened quality of life in long-term survivors [2, 3, 5–7]. The development of postoperative delirium is a result of multiple factors, including predisposing factors (such as old age, cognitive deficit, preexisting comorbidities, etc.) and precipitating factors (such as surgical trauma, pain, opioids, and surgery-related stress response) [8].

Early diagnosis is essential for delirium prevention and treatment [8]. Unfortunately, underestimation and misdiagnosis of delirium are common and the rate may reach 50 to 70% [9–11]. The DSM-5 is the gold standard for diagnosing delirium [1]. However, the criteria of DSM-5 lack practical and standardized assessment methods for attention, awareness and cognition. Its proper use requires professional psychiatric background and training [12, 13]. To facilitate delirium diagnosis, several bedside assessment tools have been designed for non-psychiatric clinicians, such as the Confusion Assessment Method (CAM), the CAM for the Intensive Care Unit (CAM-ICU) and the 3-Minute Diagnostic Interview for CAM-defined Delirium (3D-CAM) [14–16]. Of these, the CAM is the most widely used delirium assessment tool, but it still has a defect in inconsistence of diagnostic criteria and substantial training is required to guarantee the quality of assessment [14, 17]. To overcome this defect, the 3D-CAM is derived from the CAM and reconstructed with definite criteria for administration [16]. In its original validation study, the 3D-CAM performs well in elderly patients with high sensitivity (93 to 96%) and specificity (86 to 96%) [16].

The aim of this study was to translate the 3D-CAM into Chinese and to validate the 3D-CAM Chinese version (3D-CAM-CN) in surgical patients.

## Methods

This was a prospective diagnostic study to validate the reliability of the 3D-CAM-CN in surgical patients. The study protocol was approved by the Clinical Research Ethics Committee of Peking University First Hospital (2017–1321) and registered on Chinese Clinical Trial Registry on July 6, 2017 (http://www.chictr.org.cn, ChiCTR-OOC-17011887). The study was administrated in Peking University First Hospital. Written informed consents were obtained from all enrolled patients or their surrogates.

### Translation and back translation

After approval by Dr. Edward R. Marcantonio, [16] translation and back-translation were performed according to the principles of translation and cultural adaptation of patient report outcome measures [18, 19]. Firstly, the original 3D-CAM was translated into Chinese by two anesthesiologists (DLM and DXW) and one psychiatrist (XYS) independently. The three translational versions then were discussed and merged into a final version. Back translation was performed in regardless of any information from its original version. Both the translated and back translated versions (Supplement 1) were sent to Dr. Marcantonio for approval.

To be noted, the translation of items 6 and 7 was adapted according to Chinese culture (Supplement 2). In Chinese, answers to the original items 6 and 7 will be “xingqi (week) 6, xingqi 5, … , and xingqi tian (week day)” and “month 12, month 11, … , and month 1” respectively. This means there are only numeric changes of the word sequence and the difficulty of the tests will be decreased. After discussion with a panel of psychiatrists and approval from Dr. Marcantonio, we adopted “seasons backward” and “minus calculation” instead of the original item 6 and 7, respectively (Supplement 2). Both the recall of seasons in a backward sequence and the minus calculation have been used to test attention in several psychometric instruments and have been validated in Chinese population [20–22].

### Participants

The inclusion criteria were adult patients (age ≥ 18 years old) who were admitted to the ICU after surgery with a predicted length of stay for more than 48 h. Those who met any of the following criteria were excluded: (1) refused to participate; (2) history of dementia, schizophrenia, epilepsy, Parkinsonism, or any other cerebral disease that might impede communication; (3) hearing/vision impairment, language barrier or endotracheal intubation which might impede communication; or (4) coma or deep sedation.

### Enrollment and baseline data collection

One day before surgery, researchers visited patients who met the inclusion and exclusion criteria. Study protocol and related procedures were explained thoroughly to potential participants. After obtaining written informed consent, baseline data were collected which included demographic characteristics, current diagnosis and history of comorbidities. Cognitive function was assessed with the Mini-Mental State Examination (MMSE, total range 0–30, with higher score indicating better cognitive function) [22]. Patients with a MMSE score of less than 27 were considered to have preoperative cognitive impairment [22, 23].

### Delirium assessment

#### Delirium assessment with the 3D-CAM-CN

Before the study period, all researchers participated in a 3-h training program on delirium and delirium assessment. The theoretical lecture session included the clinical manifestations and diagnosis of delirium, the structure and content of the 3D-CAM-CN, as well as the key points in administrating the 3D-CAM-CN. In the practical training session, two interviewers (PPD and MJL, anesthesia residents) firstly administered the instrument to each other and then to actual patients; the training continued until the diagnosis of delirium reached 100% agreement with the panel of psychiatrists.

During the study period, the two interviewers (Role A [PPD] and Role B [MJL]) were designated to assess delirium with the 3D-CAM-CN. To reduce bias, random numbers were created by a biostatistician to determine the sequence of assessment (i.e., A-B or B-A). The first interviewer asked question at each time during the assessment; the two interviewers then completed delirium assessment independently and were blinded to each other’s result. Patients were consecutively followed-up and repeatedly assessed for delirium from postoperative days 1 to 3 between 18:00 and 20:00. All assessment processes were recorded by video.

#### Delirium assessment with the DSM-5

One of the psychiatrist panel members (SZZ, QG and QT), who was blinded to the 3D-CAM-CN assessment results, evaluated the patients according to the criteria of DSM-5 within 3 min after the 3D-CAM assessment [1]. The interview process was also recorded by video. The panel then reviewed the video together and made a decision if the patient suffered from delirium. All diagnostic results were reconfirmed by a consultant psychiatrist (XYS). The final results were considered as the reference standards.

#### Validation of adapted items 6 and 7

Each time during delirium assessment, the psychiatrist panel also made a special examination of attention and judged if the patient had inattention. This was considered as the reference standard for adapted items 6 and 7. The sensitivity and specificity were calculated to test the diagnostic characteristics of these adapted items.

### Statistical analysis

#### Sample size calculation

In our previous study, the incidence of delirium after noncardiac surgery was about 15% [24]. We assumed that the width of confidence interval was 0.2; with the sensitivity and specificity set at 90%, we need to enroll 230 and 41 patients, respectively. Considering a 5% loss to follow-up rate, we planned to enroll 245 patients.

#### Outcome analysis

Continuous data was presented as mean (standard deviation, SD) or median (interquartile range). Categorical data was presented as number (percentage). The sensitivity and specificity were calculated to analyze the diagnostic characteristics of 3D-CAM-CN in comparison with the reference standard. The sensitivity and specificity of adapted items 6 and 7 were also calculated according to the reference standard. *Kappa* coefficient was employed to analyze the interrater reliability between two interviewers. All statistical analyses were performed with SPSS 25.0 (IBM SPSS Inc., Chicago, IL, USA). Two-tailed *P* value of less than 0.05 was considered statistically significant.

## Results

### Patients

**Fig. 1:**
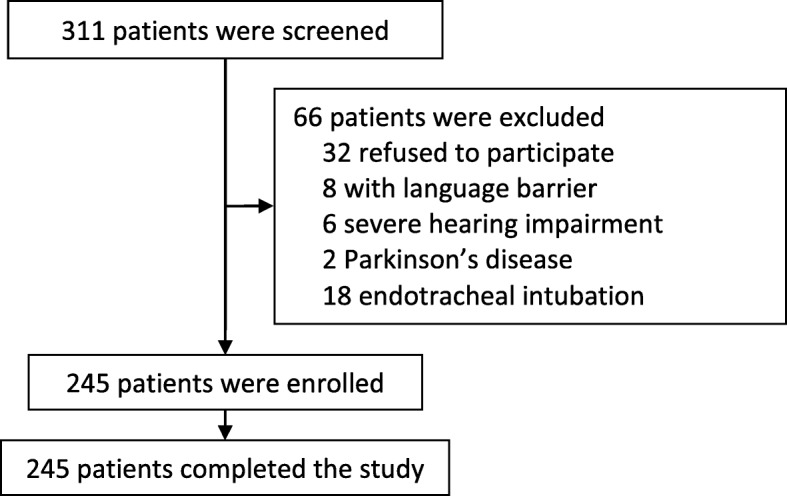
Flowchart of present study

From July 19, 2017 to September 10, 2017, 293 patients were screened. Of these, 245 gave written informed consents and were enrolled; all these patients completed the study and were included in final analyses (Fig. 1). The enrolled patients had a mean age of 73.0 (SD 10.0) years; 22.0% of them (54/245) had preoperative cognitive impairment. Baseline data were presented in Table 1.

**Table 1:** Baseline and perioperative data

Variables	Enrolled patients (***n*** = 245)
Age, year	73.0 (10.0)
Female gender	92 (37.6)
Height, cm	165.6 (8.3)
Body weight, kg	65.9 (11.3)
Previous medical history	
Stroke	23 (9.4)
Hypertension	142 (58.0)
Coronary heart disease	81 (33.1)
Arrhythmia	31 (12.7)
Pneumonia	1 (0.4)
COPD	9 (3.7)
Asthma	5 (2.0)
Diabetics	65 (26.5)
Hyperlipidemia	2 (0.8)
Chronic renal failure	14 (5.7)
Hypothyroidism	5 (2.0)
Hyperthyroidism	2 (0.8)
MMSE, score	27.5 (2.8)
MMSE < 27	54 (22.0)
Type of surgery	
Gastrointestinal	67 (27.3)
Thoracic	22 (9.0)
Orthopedic	45 (18.4)
Urological	111 (45.3)
APACHE II, score	12.5 (4.3)

Data was presented as mean (standard deviation) or number (percentage)

*COPD* chronic obstructive pulmonary disease; *MMSE* Mini-Mental State Examination; *APACHE* Acute Physiology and Chronic Health Evaluation

### Detection of delirium

Each patient was assessed for at least two consecutive days, resulting 647 paired assessments. According to the DSM-5, the psychiatrist panel reported that 9.8% (24/245) of patients suffered from at least one episode of delirium during the interview assessment. The incidence of delirium reported by Roles A and B was 12.7% (31/245) and 12.2% (30/245), respectively.

### Validation of the 3D-CAM-CN assessment

When compared with the reference standard, the sensitivity and specificity of the 3D-CAM-CN assessment by Role A were 87.2% (95% CI 76.7 to 97.7%) and 96.7% (95% CI 95.3 to 98.1%), respectively; and those by Role B were 84.6% (95% CI 73.3 to 95.9%) and 97.4% (95% CI 96.1 to 98.6%), respectively (Table 2). There was a good interrater reliability between Roles A and B (*Kappa* coefficient = 0.820, *P* < 0.001).

**Table 2:** Validation of the 3D-CAM-CN assessment

Reference standard by DSM-5	Role A			Role B		
	Delirium (yes)	Delirium (no)	Total	Delirium (yes)	Delirium (no)	Total
**Delirium (yes)**	34	5	39	33	6	39
**Delirium (no)**	20	588	608	16	592	608
**Sensitivity**	87.2% (76.7–97.7%)			84.6% (73.3–95.9%)		
**Specificity**	96.7% (95.3–98.1%)			97.4% (96.1–98.6%)		
***Kappa***	0.820					
***P*value**	< 0.001					

Results are presented as number and estimate (95% confidence interval)

### Exploratory analysis of validation of the 3D-CAM-CN assessment

In all enrolled patients, 264 paired assessments were completed in the ICU and 383 in the general ward. The 3D-CAM-CN performed well in both settings with the sensitivity ranged from 82.6 to 93.8% and the specificity from 96.7 to 98.1%, respectively (Table 3).

**Table 3:** Exploratory analysis of validation of the 3D-CAM-CN assessment

**Reference standard by the DSM-5**	**In the IC**U						**In the general ward**					
	**Role A**			**Role B**			**Role A**			**Role B**		
	**Delirium** **(yes)**	**Delirium** **(no)**	**Total**	**Delirium** **(yes)**	**Delirium** **(no)**	**Total**	**Delirium (yes)**	**Delirium** **(no)**	**Total**	**Delirium** **(yes)**	**Delirium** **(no)**	**Total**
**Delirium (yes)**	19	4	23	19	4	23	15	1	16	19	4	23
**Delirium (no)**	8	233	241	9	232	241	12	355	367	9	232	241
**Sensitivity**	82.6% (67.1–98.1%)			82.6% (67.1–98.1%)			93.8% (81.9–100%)			87.5% (71.3–100%)		
**Specificity**	96.7% (94.4–98.9%)			96.3% (93.9–98.7%)			96.7% (94.9–98.5%)			98.1% (96.7–99.5%)		
***Kappa*coefficient**	0.817						0.822					
***P*value**	< 0.001						< 0.001					
**Reference standard by the DSM-5**	**In patients with normal cognition**						**In patients with cognitive impairment**					
	**Role A**			**Role B**			**Role A**			**Role B**		
	**Delirium (yes)**	**Delirium (no)**	**Total**	**Delirium (yes)**	**Delirium (no)**	**Total**	**Delirium (yes)**	**Delirium (no)**	**Total**	**Delirium (yes)**	**Delirium (no)**	**Total**
**Delirium (yes)**	28	4	32	26	6	32	6	1	7	7	0	7
**Delirium (no)**	14	456	470	11	459	470	6	132	138	5	133	138
**Sensitivity**	87.5% (76.0–99.0%)			81.3% (67.7–94.8%)			85.7% (59.8–100%)			100% (100–100%)		
**Specificity**	97.0% (95.5–98.6%)			97.7% (96.3–99.0%)			95.7% (92.2–99.1%)			96.4% (93.3–99.5%)		
***Kappa*coefficient**	0.849						0.727					
***P*value**	< 0.001						< 0.001					

Results are presented as number and estimate (95% confidence interval)

In 191 patients with normal cognitive function, 502 paired assessments were completed. The sensitivity of 3D-CAM-CN assessment ranged from 81.3 to 87.5%, and the specificity from 97.0 to 97.7%. In 54 patients with cognitive impairment, 145 paired assessments were completed. The sensitivity of 3D-CAM-CN assessment ranged from 85.7 to 100%, and specificity from 95.7 to 96.4% (Table 3).

### Validation of the assessment with adapted items 6 and 7

After culture adaptation, items 6 and 7 showed acceptable sensitivity and specificity in validation test (for item 6: sensitivity 71.7 to 73.9%, specificity 78.0 to 78.9%; for item 7: sensitivity 82.6%, specificity 64.9 to 65.1%). The *Kappa* coefficient of interrater reliability was 0.943 and 0.822, respectively (all *P* < 0.001) (Table 4).

**Table 4:** Validation of adapted items 6 and 7 assessment

**Reference standard by psychiatrists**	**Item 6 (seasons of year, backward)**						**Item 7 (minus calculation)**					
	**Role A**			**Role B**			**Role A**			**Role B**		
	**Inattention (yes)**	**Inattention (no)**	**Total**	**Inattention (yes)**	**Inattention (no)**	**Total**	**Inattention (yes)**	**Inattention (no)**	**Total**	**Inattention (yes)**	**Inattention (no)**	**Total**
**Inattention (yes)**	33	13	46	34	12	46	38	8	46	38	8	46
**Inattention (no)**	127	474	601	132	469	601	211	390	601	210	391	601
**Sensitivity**	71.7% (58.7–84.8%)			73.9% (61.2–86.6%)			82.6% (71.7–93.6%)			82.6% (71.7–93.6%)		
**Specificity**	78.9% (75.6–82.1%)			78.0% (74.7–81.3%)			64.9% (61.1–68.7%)			65.1% (61.2–68.9%)		
***Kappa***	0.943						0.822					
***P*value**	< 0.001						< 0.001					

Results are presented as number and estimate (95% confidence interval)

## Discussion

Our results showed that the 3D-CAM-CN can be used as a reliable instrument for delirium assessment in patients after surgery. It performed well in patients with mild cognitive impairment or in the ICU settings without endotracheal intubation.

More than 28 kinds of assessment instruments are constructed and introduced to facilitate delirium screening [17, 25]. These instruments significantly improved the efficacy and accuracy of delirium diagnosis. Of these, the CAM was proposed to be the most efficient one [17, 25]. However, one deficit of the CAM is that there exists discrepancy in diagnostic criteria between interviewers even after substantial training [17]. For example, question 2 of the CAM is designed to access if a patient experiences inattention; but there are no clear criteria and predefined assessment method [26]. The interviewer’s skill of interrogation will significantly influence assessment result [17]. The 3D-CAM is derived from the CAM and provides a brief and structured assessment algorithm to accelerate and simplify the process of diagnosis [16].

The present study validated the efficacy and accuracy of the 3D-CAM-CN in surgical patients in both the ICU setting (without endotracheal intubation) and the general ward. Several bed-side instruments have been used for diagnosing delirium in ICU patients, such as the CAM-ICU and the Intensive Care Delirium Screening Checklist (ICDSC) [27]. However, these instruments are seldom compared with the 3D-CAM in ICU patients. In a prospective cohort study of 101 non-ICU geriatric patients (aged ≥75 years), the authors reported that the 3D-CAM was superior than the CAM-ICU in diagnosing delirium [28].

Delirium assessment in patients with cognitive impairment or dementia is a challenging task [29]. Lack of an efficient instrument is considered the main reason of mis- and underdiagnosis of delirium in this population [29, 30]. Marcantonio and colleagues [16] reported that the sensitivity and specificity of the 3D-CAM in dementia patients was 96% and of 86%, respectively. Our study also confirmed that the 3D-CAM-CN performed well in patients with mild cognitive impairment, with high sensitivity (85.7 to 100%) and specificity (95.7 to 96.4%).

Inattention is considered as a core characteristic of delirium by the DSM-5 [1]. Backward recall of weekdays and months is used to detect inattention in the original version of the 3D-CAM. However, in Chinese, answers to the original items 6 and 7 are too simple to guarantee their sensitivity and specificity. In the present study, two alternative tools were adopted for inattention assessment from the MMSE and the Loewenstein Occupational Therapy Cognitive Assessment Battery-second edition [20–22]. In our results, the adapted items 6 and 7 showed good sensitivity and specificity in detecting inattention with high interrater reliability.

The strength of our study included the sufficient pre-study training, the strict reference standards provided by a panel of psychiatrists, and the sufficient sample size. However, limitations also exist. The present study was performed in a single center and only enrolled patients after surgery. These may limit the generalizability of our results.

## Conclusions

Our study confirmed that the 3D-CAM-CN can be used as a reliable and accurate instrument for delirium assessment in surgical patients. It performs well in non-intubated patients in the ICU and in those with mild cognitive impairment.

## Supplementary information

**Additional file 1.** Supplement 1 Back translation of 3D-CAM Chinese version

**Additional file 2.** Supplement 2. Culture adaptation of items 6 and 7

## Data Availability

The datasets used and/or analyzed during the current study are available from the corresponding author on reasonable request.

## Abbreviations

3D-CAM: 3-Minute Diagnostic Interview for CAM-defined Delirium
3D-CAM-CN: 3-Minute Diagnostic Interview for CAM-defined Delirium Chinese version
ICU: Intensive care unit
DSM-5: Diagnostic and Statistical Manual of Mental Disorders-fifth edition
CAM: Confusion Assessment Method
CAM-ICU: CAM for the Intensive Care Unit
MMSE: Mini-Mental State Examination
ICDSC: Intensive Care Delirium Screening Checklist
SD: Standard deviation
CI: Confidence interval
