# Supplementary material for: Cross-cultural adaptation and validation of the 3D-CAM Chinese version in surgical ICU patients
Source: BMC Psychiatry. 2020 Mar 24;20:133. doi: 10.1186/s12888-020-02544-w (PMC7092439; doi:10.1186/s12888-020-02544-w)
Supplement: Supplementary file 1 — Additional file 1. Supplement 1 Back translation of 3D-CAM Chinese version [file 12888_2020_2544_MOESM1_ESM.docx]

**Supplement 1 Back translation of 3D-CAM Chinese version**

**3D-CAM (For research: version 4.1)**

**Evaluator:**

**Date:**

**Patients:**

**Time:**

**COGNITIVE FUNCTION:**

**Now I will ask you several questions to test your memory. Please do not worry if you do not know the answers.**

*[Evaluator can repeat each question once]*

*(Please record answers of each question and circle the number as indicated)*

| **Number** | **ORIENTATION** | **Correct** | **Error** | **REF** | **No response** |
| --- | --- | --- | --- | --- | --- |
| 1 | Please tell me what year we are in now? | 1 | 2 | 7 | 8 |
| 2 | Please tell me what day is today of the week? | 1 | 2 | 7 | 8 |
| 3 | Please tell me where you are? [eg. hospital] | 1 | 2 | 7 | 8 |

*****If response to any of the 3 items is not “correct”, feature 3 is present.***

**DIGITAL SPAN**

*[Read digits at a rate of one per second]*

Now I am going to read some numbers, please repeat the numbers in a reverse direction. For example, if I read 6-4, you will say 4-6.

| **Number** | | **Digital backward** | | **Response** | **Correct** | **Error** | **REF** | **No response** |
| --- | --- | --- | --- | --- | --- | --- | --- | --- |
| 4 | 7-5-1 | | __-__-__ | | 1 | 2 | 7 | 8 |
| 5 | 8-2-4-3 | | __-__-__-__ | | 1 | 2 | 7 | 8 |

**6. SEASONS OF YEAR BACKWARD**

Please tell me the seasons of the year backward, beginning with winter.

*(Can prompt once like this: “what is the season before winter? If patient tell seasons in a forward direction, please repeat the overall instructions)*

| Season | Response | Correct | Error | REF | No response |
| --- | --- | --- | --- | --- | --- |
| Winter |  | 1 | 2 | 7 | 8 |
| Autumn |  | 1 | 2 | 7 | 8 |
| Summer |  | 1 | 2 | 7 | 8 |
| Spring |  | 1 | 2 | 7 | 8 |
| *Record each response in sequence.*  *Coding Instructions: An error on any season makes the entire item incorrect.* | | | | | |

**7. CALCULATION**

Now you are going to do minus calculation, starting from 20, minus 3 each time, please continue calculation until I say stop.

(If the subject stops at X, evaluator can prompt once like this: “what is the result of X minus 3?”)

| Subtraction | Response | Correct | Error | REF | No response |
| --- | --- | --- | --- | --- | --- |
| 20-3 |  | 1 | 2 | 7 | 8 |
| 17-3 |  | 1 | 2 | 7 | 8 |
| 14-3 |  | 1 | 2 | 7 | 8 |
| 11-3 |  | 1 | 2 | 7 | 8 |
| 8-3 |  | 1 | 2 | 7 | 8 |
| *Record each response in sequence.*  *Coding Instructions: If the subject leaves 1 result out or makes 1 error, the entire item is incorrect.* | | | | | |

*****If any response of items 4, 5, 6, 7 are not “correct”, feature 2 is present.***

**PATIENT REPORTED SYMPTOMS:**

*If the answer of any of the following questions is “YES”, please inquire and record details. For example, the frequency.*

*If the answer is nonsensical, code as 8.*

I am going to ask you some questions about how you have been thinking during the past day.

**8. Have you felt confused during the past day?** *(about basic information, such as orientation, reasons of hospitalization, not details of medical condition or treatment)*

1-No 2-Yes 7-REF 8-Nonsensical 9-NA

**9. Did you think you were not really in hospital during the past day？**

1-No 2-Yes 7-REF 8-Nonsensical 9-NA

**10. Did you see any things that were not really there during the past day?** *(If patient is blind skip and code 9)*

1-No 2-Yes 7-REF 8-Nonsensical 9-NA

***** If any response of items 8, 9, 10 are anything other than ’no’, feature 1 is present.***

**End of interview. Thanks for your cooperation!**

**Part 2**

Please go to next step to complete interview observation items and final coding.

Used with permission. SAGES study: The SAGES Study: Training Manual and Questionnaires; 2010; Boston, Aging Brain Center.

| **Number** | **OBSERVATIONS** | **Result** |
| --- | --- | --- |
| **11A** | **Was the patient sleepy during the interview?** (requires the patient actually fall asleep, but can be aroused easily) | IS FEATURE 4 PRESENT？  1-No 2-Yes |
| **11B** | **Was the patient stuporous or comatose during the interview?** (Difficult or impossible to arouse) | IS FEATURE 4 PRESENT？  1-No 2-Yes |
| **12** | **Did the patient show hypervigilance?** (having excessively strong responses to ordinary objects/stimuli in the environment, or being inappropriately startled, etc.?) | IS FEATURE 4 PRESENT？  1-No 2-Yes |
| **13** | **Was the patient’s thinking unclear and illogical?** (nonsensical speech, inappropriate answers to questions, contradictory statements or shifting unpredictably from one subject to another) | IS FEATURE 3 PRESENT？  1-No 2-Yes |
| **14** | **Was the patient's conversation rambling, inappropriately verbose, or no relation with subject?** (off-target conversation or telling a story unrelated to the interview) | IS FEATURE 3 PRESENT？  1-No 2-Yes |
| **15** | **Was the patient's speech unusually limited or sparse?** (inappropriately brief or stereotyped answers) | IS FEATURE 3 PRESENT？  1-No 2-Yes |
| **16** | **Did the patient have trouble in keeping the same topic during the interview?** (frequently asked the interviewer to repeat questions) | IS FEATURE 2 PRESENT？  1-No 2-Yes |
| **17** | **Was the patient easily attracted by environmental stimuli?** (such as television, people outside the room, roommate's conversations) | IS FEATURE 2 PRESENT？  1-No 2-Yes |
| **18** | **Was there any fluctuation of the patient's level of consciousness during the interview?** (frequently fell alseep for part of the interview, but was completely awake for part of the interview) | IS FEATURE 1 PRESENT？  1-No 2-Yes |
| **19** | **Was there any fluctuation of the patient's level of attention during the interview?** (very inattentive for part of the interview, but attentive for part of the interview-- Note: just answer some questions correctly and others incorrectly does support this feature) | IS FEATURE 1 PRESENT？  1-No 2-Yes |
| **20** | **Was there any fluctuation of the patient's speech/thinking during the interview?** (speaks very slowly during part of the interview and then very fast, or speech was coherent for part of the interview and then nonsensical) | IS FEATURE 1 PRESENT？  1-No 2-Yes |

**CAM-summary FEATURES 1-4**

| **Coding for features 1-4 (0) No, (1) Yes** | | | |
| --- | --- | --- | --- |
| ___ | **1** | **Acute Onset and Fluctuating Course** | If any of the items 8, 9, 10, 18, 19, 20 is “incorrect/present”, code ‘YES’ |
| ___ | **2** | **Inattention** | If any of the items 4, 5, 6, 7, 16, 17 is “incorrect/present”, code ‘YES’ |
| ___ | **3** | **Disorganized Thinking** | If any of the items 1, 2, 3, 13, 14 is “incorrect/present”, code ‘YES’ |
| ___ | **4** | **Altered Level of Consciousness** | If any of the items 11 and 12 is “present”, code ‘YES’ |

**Only when Feature 1 is not present, but Feature 2 and either Feature 3 or Feature 4 is present, the following evaluation need to be completed.**

| **Number** |  |  |
| --- | --- | --- |
| **21** | **IF IT IS THE FIRST DAY OF HOSPITALIZATION AND THERE IS NO PREVIOUS 3D-CAM ASSESSMENT RESULTS:** Review the medical record or contact a family member, friend, or health care provider who knows the patient well to find out if the patient is experiencing an acute change. "Is the patient experiencing an acute change in their memory or thinking?" | IS FEATURE 1 PRESENT？  1-No 2-Yes* 9-Skip |
| **22** | **IF IT IS THE SECOND DAY OF HOSPITALIZATION OR LATER AND PREVIOUS 3D-CAM RATINGS ARE AVAILABLE:** Review previous 3D-CAM assessments and determine if there has been an acute change in performance, based on ANY new "positive" items | IS FEATURE 1 PRESENT？  1-No 2-Yes* 9-Skip |

****If the answer is “yes”, go back and score CAM Feature 1.**

**The diagnosis of delirium by CAM requires the presence of Feature1 + Feature 2 + either Feature 3 or 4.**

**____ 5. Delirium present? (0) No (1) Yes**

**CAM Copyright 2003, Hospital Elder Life Program, LLC. Not to be reproduced without permission**
