# Supplementary material for: Cross-cultural adaptation and validation of the 3D-CAM Chinese version in surgical ICU patients
Source: BMC Psychiatry. 2020 Mar 24;20:133. doi: 10.1186/s12888-020-02544-w (PMC7092439; doi:10.1186/s12888-020-02544-w)
Supplement: Supplementary file 2 — Additional file 2. Supplement 2. Culture adaptation of items 6 and 7 [file 12888_2020_2544_MOESM2_ESM.docx]

**Supplement 2. Culture adaptation of items 6 and 7**

| **Original version and direct translation into Chinese** |  |
| --- | --- |
| **Item 6. DAYS OF WEEK BACKWARDS**  Please tell me the days of the week backwards, starting with Saturday.  *Answer example: Saturday, Friday, Thursday, Wednesday, Tuesday, Monday, Sunday* | **6. 倒数日期**  请从星期六开始倒数星期。  *回答示例：星期6，星期5，星期4，星期3，星期2，星期1，星期日* |
| **Item 7. MONTHS OF YEAR BACKWARDS**  Please tell me the months of the year backwards, starting with December.  *Answer example: December, November, October, ……, March, February, January* | **7. 月份倒数：**  请倒数月份，从12月开始。  *回答示例：12月，11月，10月，……3月，2月，1月* |
| **Culture adaptation of translation and back translation** |  |
| **Item 6. SEASONS OF YEAR BACKWARD**  Please tell me the seasons of the year backward, beginning with winter.  *Answer example: Winter, Autumn, Summer, Spring* | **6. 季节倒数**  请从冬季开始，倒着说出季节。  *回答示例：冬季、秋季、夏季、春季* |
| **Item 7. MINUS CALCULATION**  Now you are going to do minus calculation, starting from 20, minus 3 each time, please continue calculation until I say stop.  *Answer example: 20-3=? 17-3=? 14-3=? 11-3=? 8-3=?* | **7.计算**  下面需要您做一项减法运算，从20开始，每次减去3，请连续计算，直到我说停为止。  *回答示例： 20-3=？再减3=？，再减3=？，再减3=？，再减3=？* |
